# When gender matters: parental characteristics associated with children’s compliance with 24-hour movement behavior guidelines – the Czech FAMIPASS study

**DOI:** 10.1186/s12889-025-25497-9

**Published:** 2025-11-27

**Authors:** Dagmar Sigmundová, Jaroslava Voráčová, Jan Dygrýn, Michal Vorlíček, Erik Sigmund

**Affiliations:** 1 Institute of Active Lifestyle, Faculty of Physical Culture, Palacký University Olomouc, třída Míru 117, Olomouc, 771 11 Czechia; 2 Department of Social Sciences in Kinanthropology, Faculty of Physical Culture, Palacký University Olomouc, třída Míru 117, Olomouc, 771 111 Czechia

**Keywords:** Sedentary behavior, Physical activity, Sleep, Daughter, Son, Mother, Father

## Abstract

**Background:**

Family is important for the support and promotion of healthy movement behaviors of children. Therefore, the aim of this study was to identify parental characteristics that are associated with children’s adherence to the World Health Organization (WHO) guidelines on 24-hour movement behavior (24hMB), based on accelerometer data from the daily lives of both parents and their families.

**Methods:**

The 24-hour movement behavior (24hMB) of 217 family triads was continuously monitored over a 7-day period using ActiGraph accelerometers placed on the non-dominant wrist of each participant as part of the Czech FAMIPASS study. Children’s compliance with the WHO 24hMB guidelines (sleep, sedentary behavior, physical activity) was analyzed using backward logistic regression analysis separately for the daughter-mother-father and son-mother-father models.

**Results:**

Without significant gender differences, 25.2%_DAUGHTERS_ and 26.7%_SONS_ (or 71.7%_DAUGHTERS_ and 78.1%_SONS_) met all 3 (or a combination of ≥ 2) 24hMB guidelines, although the contributions of mothers and fathers differed. For daughters only, fathers’ overweight/obesity significantly reduced the chances of achieving the recommended amount of sedentary behavior/a combination of ≥ 2 24hMB guidelines (*p* = 0.03/0.003). Mother’s overweight/obesity significantly reduced the odds ratio of meeting the recommended amount of sedentary behavior for both daughters/sons (*p* = 0.04/0.002), achieving the recommended amount of moderate-to-vigorous physical activity in daughters (*p* = 0.03), and meeting a combination of ≥ 2 24hMB guidelines in sons (*p* = 0.03). Maternal university education significantly contributed to achieving the recommended sedentary behavior in both daughters/sons (*p* = 0.03/0.02) and to fulfilling a combination of ≥ 2 24hMB guidelines in daughters (*p* = 0.04).

**Conclusions:**

Mothers’ non-excessive body weight, higher level of education and meeting ≥ 2 WHO 24hMB recommendations, compared with those of fathers, are significantly associated with children’s adherence to the WHO 24hMB guidelines in two-parent households. It suggests that mothers play an important role in shaping the daily 24hMB that supports their children’s health.

## Background

Sleep, sedentary behavior (SB) and physical activity (PA) together shape our 24-hour movement behavior (24hMB), and maintaining an appropriate balance of these components contributes to overall health [1, 2]. Particularly for children, the family is a key source of influence that can affect their 24hMB [3]. The family can support children and adolescents in achieving healthy PA, SB and sleep behaviors by encouraging, facilitating, modeling, setting expectations and actively engaging in these behaviors with them [3]. Parents also serve as gatekeepers in deciding which activities children will engage in and what resources and access, they have available [4]. Nowadays, however, there are many types of families and caregiving arrangements, which can be generally categorized into single-parent households (single parenting) and two-parent households (couple parenting). Based on this classification, it was found that 5–11-year-old children from couple-parent families engaged in less SB than children from single-parent families [3]. Additionally, boys from couple-parent households had more sleep compared to boys from single-parent households, and girls from couple-parent households participated in more organized PA (OPA) than girls from single-parent households [3].

Although it has been shown previously that children benefit emotionally and socially when fathers are involved in couple parenting [5–7], later and even current studies do not distinguish parental gender in analyses of the relationship between parents and 24hMB in children [8–10], or they select only mothers as representatives of school-age children in families [11–14]. Studies that differentiated parental gender when examining associations between parents’ behavior and their children revealed that parents seemed to play a stronger role in supporting 8–9-year-old sons than daughters [15]. Also, stronger associations were observed in families where both parents shared an equal role in supporting their child [15]. Fathers’ moderate to vigorous PA (MVPA) tended to have stronger correlations with children’s MVPA compared with mothers’, as did the association of SB between parents and sons [16]. In analyses of the parent-child associations in ‘screen time’ (ST – time spent looking at an electronic device with a screen, such as a computer or television, games console, or smartphone.), SB, MVPA, and light PA on weekdays and weekends, correlations appeared to be stronger in most cases for same-sex parent-child pairs compared with opposite-sex parent-child pairs [16]. However, deeper linear regression analyses did not reveal parent gender as a significant factor in these relationships [16]. Thus, there is still a lack of valid information on the role of both parents in relation to their children’s 24hMB, and in particular, on whether they help their children meet the 24hMB guidelines [1, 17, 18].

The FAMIly Physical Activity, Sedentary behaviour and Sleep study (FAMIPASS) [19] is a scientific research response to the calls of the Czech national health and well-being related strategies “Health 2020” and “Concept of Sport Promotion 2016–2025” to add relevant information on 24hMB of preschool and school-age children. The absence of a national system to monitor PA, SB or sleep in children and adolescents has so far led to conflicting results on exercise behavior and its correlates [20]. Previous FAMIPASS studies have indicated the important role of maternal PA and sleep behaviors in helping 3–10-year-old children meet recommended PA and sleep guidelines [21, 22], including the absence of ST devices in the bedroom and a reduction in SB to achieve healthy sleep durations [22]. In addition, significant associations were found between MVPA and total PA in parent-child dyads across all gender combinations [21]. Significantly higher odds of children meeting at least two or all three of the 24hMB guidelines [1, 17, 18] were associated with mothers having a normal body weight, adherence to at least two 24hMB guidelines, and completion of more than primary education, as well as with fathers having higher levels of education and being younger in age [23]. However, all previous FAMIPASS studies [21–23] have only analyzed family relationships separately as family dyads (i.e., mother-child or father-child), rather than examining couple parenting dynamics involving the mother, father, and child simultaneously. Therefore, this study addresses a research gap in Central European countries by examining the distinct roles of mothers and fathers in supporting children’s adherence to 24hMB guidelines and by identifying previously unexamined correlates of 24hMB in children aged 3–10 years in families with both parents.

## Methods

### Study design and inclusion criteria

The FAMIPASS study is a nationally representative longitudinal study of the Czech families with preschool and younger school-age children focused on analyzing 24hMB in the context of the school and family environment [23]. Participating families were selected by stratified sampling of preschools and elementary schools from rural and urban areas of Bohemia, Moravia and Silesia to cover all permanently inhabited districts of Czechia. The first wave of data collection ran from March 21, 2022 to May 20, 2023 during the regular school week of preschools/elementary schools excluding multi-day and public holidays. All data collection took place after restrictions caused by the COVID-19 epidemic. Inclusion criteria for families in the study were:

- having at least one child of their own aged 3–10 years,
- no absence from preschool/elementary school for 1 or more days due to illness,
- regular attendance at preschool/elementary school,
- willingness to participate in the study voluntarily and free of charge, and d) providing written informed consent from parents/guardians [23].

### Participants and dataset

Of the 860 (100%) families recruited and contacted, 552 (64.2%) provided written informed consent to participate in the study and 502 (58.4%) families initiated weekly continuous 24hMB monitoring. A total of 472 (54.9%) families completed the full weekly monitoring and 217 (25.2%) family triads (mother and father with at least one child) provided valid 24hMB accelerometer data, anthropometric characteristics and questionnaire data to determine the families’ socioeconomic status (SES) and participants’ ST. Reasons for excluding 124 families from the final data set were as follows: insufficient minimum number of days of accelerometer wear, defined as at least 3 school days and 1 weekend day (*n* = 65), missing anthropometric data or data to determine families’ SES (*n* = 26), and missing data to capture children’s participation in organized forms of PA (*n* = 33). Weekly 24-hour monitoring with complete and valid 24hMB (resp. anthropometric and SES-related) data were completed by 255 (29.7%) families with at least one child aged 3–10 years. Of the 255 families, 38 were single-parent families or families with valid dyad-only data (that is, one parent-child), while 217 were couple-parent families whose anthropometric data are shown in Table 1. All statistical analyses were conducted on the final sample comprising 217 couple-parent families. Participating children and their parents were predominantly white Caucasian (>92%), which is representative of the ethnic demographics of Czechia [24]. All couple-parents’ families included in the final analysis consisted of a father (man), a mother (woman), and at least one child aged 3–10 years.

**Table 1:** Basic characteristics of families - couple parenting (mother-father-child simultaneously)

Family members	Number of participants	Calendar age M/SD	Height (cm) M/SD	Weight (kg) M/SD	BMI (kg/m^2^) M/SD
Daughters	*N* = 104	75.17*/20.64	119.46/12.20	22.35/6.11	15.42/2.15
Sons	*N* = 113	77.76*/19.48	120.58/11.60	22.59/5.11	15.39/1.63
Mothers	*N* = 217	36.99^#^/3.97	167.51/6.18	65.88/11.32	23.47/3.84
Fathers	*N* = 217	39.94^#^/5.22	181.10/6.99	85.23/12.82	26.00/3.59

*N* Number, *M* Mean, *SD* Standard deviation, */^#^ – indicates calendar months/years, *cm * centimeters, *kg* kilograms, *BMI* Body Mass Index (kg/m^2^)

### Anthropometric characteristics, socioeconomic status and ‘screen time’ of participants

During a joint meeting involving researchers, parents and preschool/elementary school administrators and teachers, the researchers presented the study design and detailed procedures. These included instructions for measuring body weight and height at home using graphic instructions, the method of attaching and wearing the accelerometer on the non-dominant wrist, and recording time data in the family diary [19, 21, 22]. The researchers also explained how to record ST and SES data to the family diary [19, 21, 22], as well as the process for delivering individual feedback using graphic sheets for each participant.

Parents were instructed to measure their own and their children’s body weight and height in the morning before breakfast, while wearing underwear. Parents recorded body weight/height to the nearest 0.1 kg/0.5 cm [25]. Parents’ measurement of their children’s body weight and height in the home environment has been confirmed to be sufficiently valid for calculating body mass index (BMI) and subsequent detection of excess body weight in 4–10-year-old children [26, 27].

Family SES was measured by the summary scores of responses to six questions about the family’s material background that were part of the family diary [19, 21, 22]. The content of each question (with categorized response options) was as follows: having one’s own bedroom for each child in the family (0/1); number of bathrooms in the household (0/1/2/≥3); number of computers in the household (0/1/2/≥3); number of cars owned for family use (0/1/≥2); number of foreign vacations taken in the past year (0/1/2/≥3); ownership of a dishwasher in the household (0/1) [28]. According to the summary scores of the responses to the above questions, three categories of families regarding SES (low, medium, high) were identified as follows: the lowest/highest 20% of the summary scores characterized families with low/high SES, while the range of 21–79% characterized families with medium SES [23, 28]. The summary score was positively correlated with reported parental income with an Eta-squared close to 0.30. The summary test-retest reliability correlation was *r* = 0.90 (28). In the socioeconomic conditions of Czechia, the summary score was validated in relation to disposable household income (Pearson correlation *r* = 0.77, *p* < 0.001) [29].

Children’s daily ST was calculated from parents’ responses to questions about the use of screen-based devices, originally adapted from the “Health Behaviour in School-aged Children” study. Specifically, parents were asked: ‘How many hours a day do you usually spend in your leisure time on weekdays/weekends watching TV, DVDs, videos (including YouTube or similar online services)?’ and ‘How many hours a day do you usually spend in your leisure time on weekdays/weekends playing games on a computer, games console (PlayStation, Xbox etc.), smartphone, tablet or similar electronic device?’ [19, 23]. The questions were categorized based on weekdays and weekends. For each question, there were nine different response options (none/half an hour/1/2/3/4/5/6/and 7 or more hours per day). Validity and reliability of the 7-day recall questions have been verified in comparison with the 7-day 24-hour diaries for both weekdays and weekends [30]. Total ST was calculated as the sum of the weighted arithmetic means of weekday and weekend ST {weighted mean=[(average weekday×5)+(average weekend×2)]/7}.

### Accelerometer-based monitoring of 24hMB

The 24hMB was monitored using accelerometers (wGT3X-BT in children and GT9X Link in parents; ActiGraph LLC, Pensacola, FL, USA) placed on the wrist of the non-dominant hand in both parents and their offspring. To anchor the 24-hour accelerometer recording, parents recorded daily time data in a family diary, including morning wake-up, arrival at and departure from preschool/school, start and end of OPA (such as practices and coach/leader-led lessons), and bedtime. The 24-hour monitoring started at midnight on the day of the joint meeting between parents and researchers.

All accelerometers were individually initialized via ActiLife software version 6.13.4 (ActiGraph LLC, Pensacola, FL, USA) for each family member separately based on the information provided in the written informed consent. Accelerometers recorded triaxial acceleration data at a sampling rate of 100 Hz. All accelerometer data sets were analyzed using the R GGIR version 2.7-1.7.7 package, applying previously established cut-off values for participants’ 24hMB intensity. Specifically, SB was defined as acceleration values of less than 36 milligrams (m*g*); light PA as 36–200 m*g*; MVPA as 201–706 m*g*; and vigorous PA as values equal to or greater than 707 m*g* [31, 32]. The default setting for wear-free time detection in part 1 of the GGIR package in R was used. Specifically, the algorithm required that the standard deviation of the sliding window signal be close to the sensor’s noise level. If this condition was met, GGIR classified the middle 15 min of this 60-minute window as non-wear time [31]. The sleep time, i.e., the time from lying down to waking up, was determined using the default setting of a heuristic algorithm analyzing the distribution of angular changes [32]. To be included in the final data set, accelerometer data had to be observed on at least three preschool/school days and one weekend day for at least 16 h per day, and accelerometer data had to be available for each 15-min interval of the 24-h cycle [32]. Average daily sleep time, SB, light PA, MVPA, vigorous PA, and total PA were calculated as the weighted arithmetic mean of these activities performed during preschool/school and weekend days {weighted mean=[(average weekday×5)+(average weekend×2)]/7}.

### Data processing and statistical analysis

The 24hMB data along with anthropometric and sociodemographic data of the participants were analyzed using Statistical Package for the Social Sciences (SPSS) for Windows, version 26 (IBM Corp, Armonk, NY, USA). After applying the inclusion criteria, all data were checked for outliers and obvious errors and any affected cases were discarded. Calendar age was calculated as the difference between the study start date and date of birth. Participants’ BMI was calculated as the ratio of reported body weight (kg) to the square of body height (m^2^). Children with BMI z-scores >1 standard deviation (SD) and < 2 SD were classified as overweight, whereas children with BMI z-scores >2 SD were classified as obese according to the WHO reference data for that sex and age [33–35]. A BMI range of 25–29.9 kg/m^2^ (or ≥ 30 kg/m^2^) represented overweight (or obese) in parents [36]. Subsequently, the classification of participants was calculated according to the 24 MB recommendations [1, 2, 17, 18].

Family offspring aged 3–4 years with a cumulative PA of at least 180 min per day, including MVPA of at least 60 min per day, ST limited to a maximum of 1 h per day, and an average sleep duration of 10–13 h per day were classified as meeting the 24hMB guidelines for preschool children [17]. Family offspring aged 5 years and older who cumulatively performed 60 min of MVPA while having a maximum of 2 h of ST per day and sleeping 9–11 h per day met the 24hMB guidelines [1, 18]. Adult parents aged 18 years and older were classified as meeting 24hMB guidelines if their SB did not exceed 8 h per day while also realizing at least 150 min of MVPA per week and sleeping 7–9 h per day [2].

**Fig. 1:**
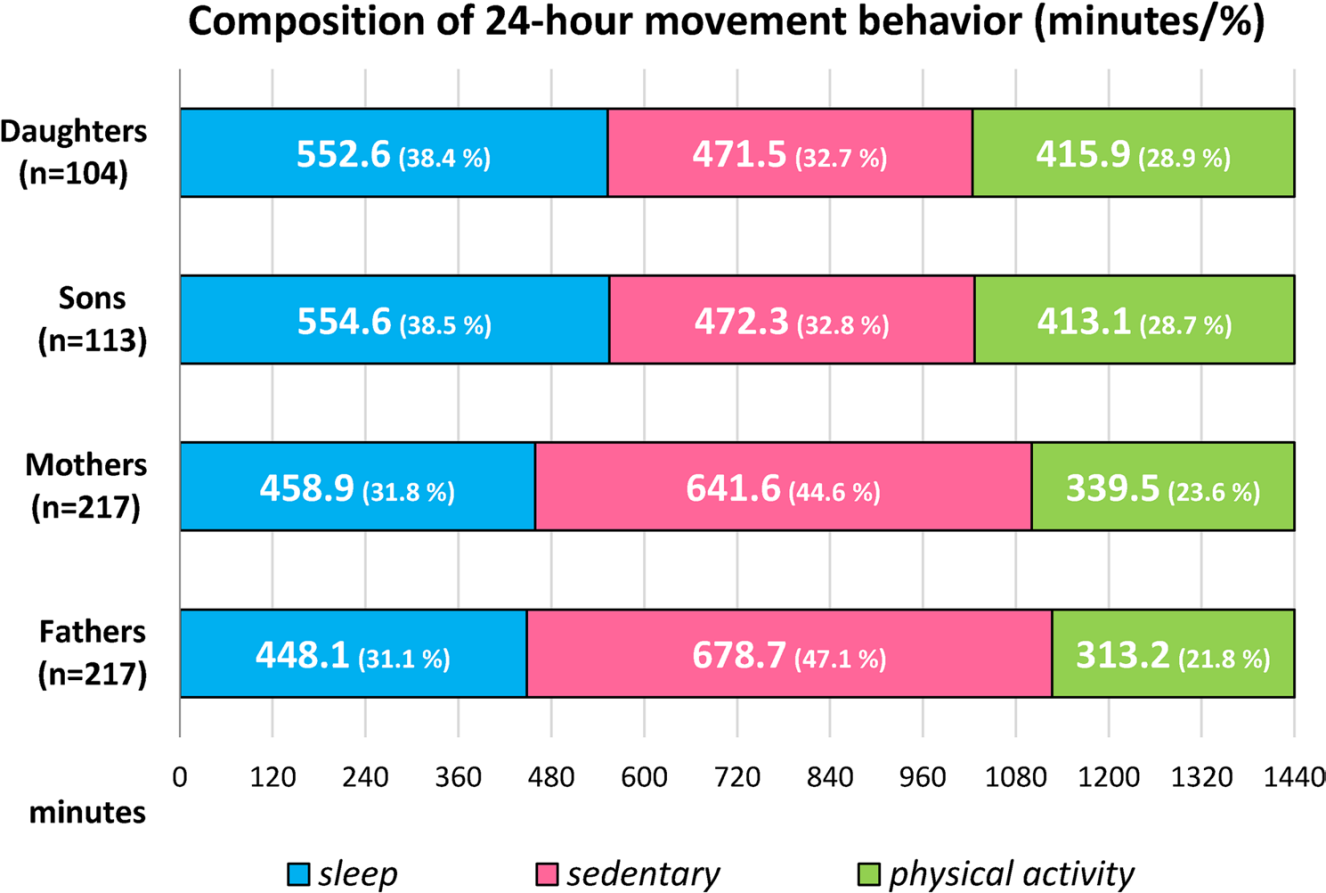
Average daily duration (minutes) and representation (%) of the components of 24hMB for family members

**Fig. 2:**
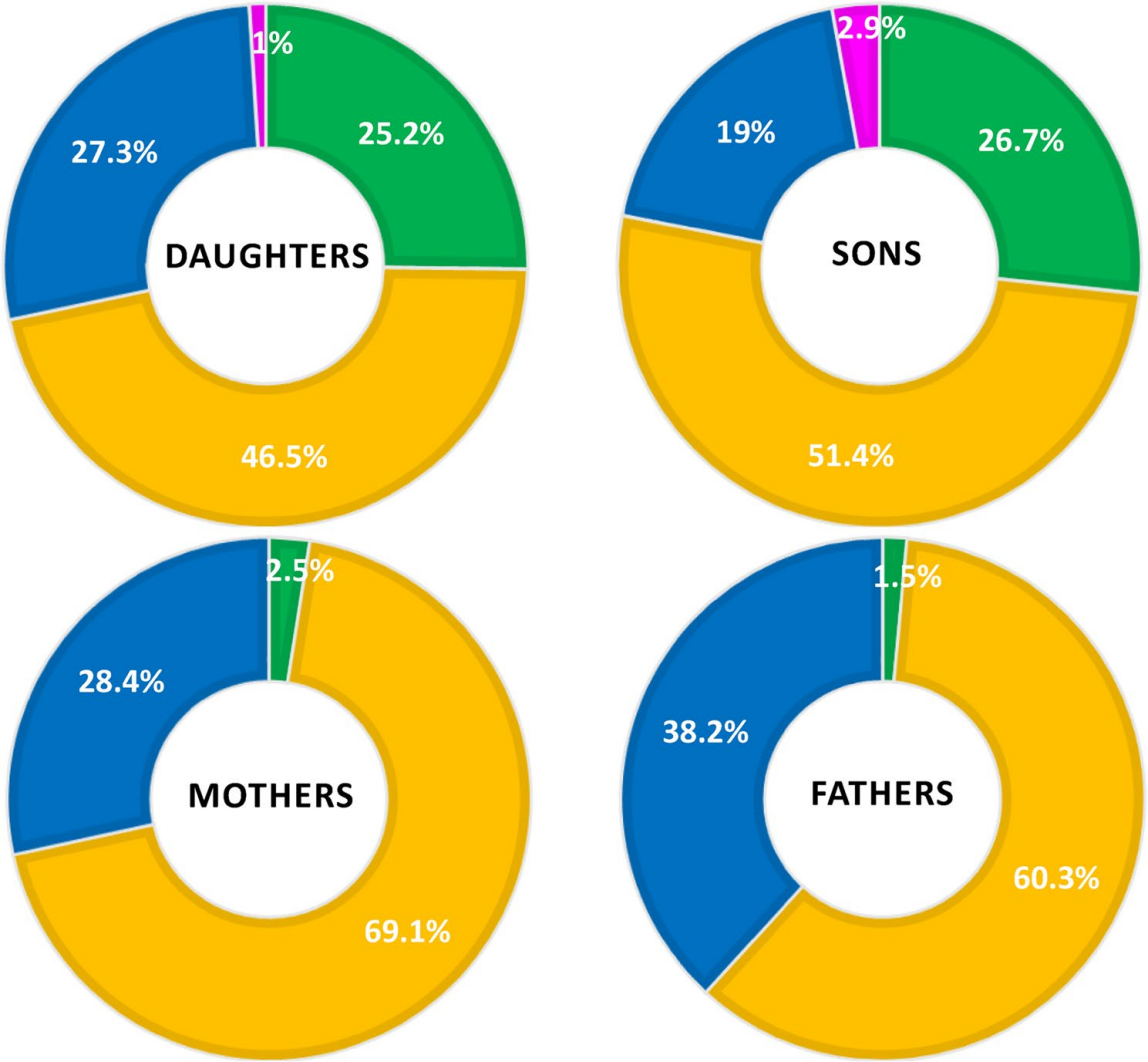
Adherence to the 24hMB guidelines of family members: all three, any combination of two, only one and none

Basic descriptive characteristics of individual family members (calendar age [months in children and years in parents], body height [cm], body weight [kg], and BMI [kg/m^2^]) are presented as arithmetic means and SDs separately for daughters, sons, mothers and fathers (Table 1). Due to the non-normal distribution of anthropometric variables, the Mann-Whitney test was used to compare calendar age, height, and weight between daughters and sons (or fathers and mothers). Pearson’s chi-square test (χ^2^) was used to examine differences in BMI between daughters and sons (or mothers and fathers). The Kolmogorov-Smirnov test confirmed the normal distribution of output variables related to 24hMB: time spent in PA, SB, and sleep. The daily duration of each component of PA, SB, and sleep during 24hMB (expressed as an arithmetic mean in minutes per day) and their respective daily percentage representation (expressed in percent) are shown in Fig. 1. Differences in daily sleep duration, SB, and PA between daughters and sons (or mothers and fathers) were determined using Univariate analysis of variance. Adherence to the 24hMB guidelines is presented as a percentage, separately for each family member (Fig. 2). The Pearson chi-square test (χ^2^) was used to test for differences in adherence to the 24hMB guidelines (separately for all three guidelines, any combination of any two guidelines, only one guideline, and none) between daughters and sons (or mothers and fathers). Associations between child and parent characteristics and children’s adherence to the 24hMB guidelines were examined using binary logistic regression Backward method in the daughter-mother-father and son-mother-father models. Analyses were conducted separately for compliance with MVPA recommendations (Model A), ST (Model B), and any combination of 2 or more 24hMB recommendations (Model C). Independent input variables were selected based on published results from the Czech FAMIPASS study [19, 21–23], which focused on parent-child dyads (mother-child or father-child). The independent input variables included the following: for children, age category (3–5.9.9/6–10 years), overweight + obesity status (no/yes), and participation in organized PA (no/yes); for parents, (mothers and fathers analyzed separately), university education (no/yes), overweight + obesity status (no/yes), and achieving the WHO 24hMB guidelines for MVPA (no/yes). SES was included as a continuous variable in all regression models. 18% of families had multiple children participating in the study, resulting in some parents being included multiple times in the analyses examining the parent–child relationship in adherence to 24hMB guidelines. The final regression models of the Backward method, Model A (Step 6), Model B (Step 6 in daughter-parents and Step 7 in son-parents), Model C (Step 6 in daughter-parents and Step 7 in son-parents), were selected to include at least one statistically significant input variable while ensuring that the model constant was not significant (Table 2). The results of the logistic regression analyses were expressed using odds ratios (ORs) and 95% confidence intervals (95% CIs). The alpha significance level was set at a minimum of 0.05.

**Table 2:** Odds ratios of children achieving the 24hMB guidelines - binary logistic regression backward method (MODEL A - MVPA guideline, MODEL B - ST guideline, MODEL C - any combination of at least two guidelines) for family triads (mother-father-child model)

		WHO’s 24hMB guidelines											
		MODEL A meeting the daily MVPA guideline				MODEL B meeting ST guideline				MODEL C meeting any combination of ≥ 2 24hMB guidelines (sleep, MVPA, ST)			
Correlating variables of family members		Daughter-parents (Step 6)		Son-parents (Step 6)		Daughter-parents (Step 6)		Son-parents (Step 7)		Daughter-parents (Step 6)		Son-parents (Step 7)	
		OR	95% CI	OR	95% CI	OR	95% CI	OR	95% CI	OR	95% CI	OR	95% CI
*Child:*													
Active participation in OPA		**2.555**	1.02/6.38	**2.858**	1.09/7.49			**2.826**	1.06/7.51	**5.409**	1.43/20.51	2.266	0.78/6.60
Overweight + obesity										5.525	0.83/36.83		
Age category								0.439	0.16/1.19				
*Family:*													
Socioeconomic status of families (SES)				**2.710**	1.12/6.53	1.530	0.68/3.46					1.217	0.46/3.24
*Mother:*													
Overweight + obesity		**0.348**	0.13/0.91			**0.326**	0.11/0.96	**0.171**	0.05/0.54			**0.284**	0.09–0.89
University education				0.401	0.14/1.14	**2.784**	1.09/7.11	**3.173**	1.23/8.17	**3.352**	1.08/10.39		
Meeting ≥ 2 WHO guidelines										**6.258**	1.69/23.22		
*Father:*													
Overweight + obesity						**0.222**	0.08/0.60			**0.237**	0.06/0.85		
University education		0.521	0.21/1.27										
Meeting ≥ 2 WHO guidelines												1.387	0.45/4.29

Results statistically significant at the p level less than 0.05 are shown in bold

Step - using the backward method, models with variables that did not have a statistically insignificant constant in the last step were selected

24hMB – 24 hour movement behavior; WHO – World Health Organization; Binary logistic regression models included independent variables for children: age category (3–5.9.9/6–10 years), overweight + obesity (no/yes), child’s participation in OPA (no/yes), and for parents (mothers and fathers separately): university education (no/yes), overweight + obesity (no/yes), achieving the WHO’s guidelines for 24-hour movement behavior (24hMB) (**A** for MVPA (no/yes), B for ‘screen time’ (no/yes), C ≥ 2 (no/yes); The SES variable was included in all regression models as a continuous variable. *PA* Physical activity, *OPA* Leisure organized *PA,* *MVPA * Moderate to vigorous physical activity, *ST * ‘Screen time’, *OR *Odds ratio (logistic regression method Backward), % percentage, *CI* Confidence interval, *p* level of significance

## Results

No statistically significant differences were found between the participating boys and girls, with a mean calendar age of 6.38 years, in any of anthropometric variables (body height, body weight, BMI - χ^2^ *p* = 0.752–0.982), in contrast to their parents, where fathers significantly (χ^2^ *p* < 0.001) outnumbered mothers in BMI (Table 1).

Sleep accounted for the longest part of the day for 3–10-year-olds living in couple families, followed by SB and PA, with no statistically significant differences (*p* = 0.891–0.992) between daughters and sons (Fig. 1). Excluding a few minutes, daughters and sons spent almost 420 min per day in PA, with sons and daughters averaging 86.4 and 73.2 min per day, respectively, in MVPA. Even the approximately 13-minute difference in daily MVPA between daughters and sons was not statistically significant (*p* = 0.11). Consistent with the results for children, we found no statistically significant differences (*p* = 0.325–0.871) between mothers and fathers in average daily sleep duration, SB, or PA. However, in contrast to children, SB represented the longest component of 24hMB for parents, averaging more than 600 min for mothers and more than 660 min per day for fathers (Fig. 1).

On average, over a quarter of the 3–10-year-old offspring (25.2% of daughters and 26.7% of sons) met all 3 of the 24hMB guidelines, and even more than two-thirds of the children (71.7% of daughters and 78.1% of sons) achieved any combination of at least 2 of the 3 24hMB guidelines with no statistically significant differences (χ^2^ *p* = 0.69) between genders (Fig. 2). For parents, daily high rates of SB resulted in very low proportions of mothers (2.5%) and fathers (1.5%) meeting all 3 24hMB guidelines. The 8.8% difference between mothers and fathers who achieved any combination of 2 of the 3 24hMB guidelines (69.1% vs. 60.3%) was marginally significant (*p* = 0.0551) according to the χ^2^ test (Fig. 2). A negligible percentage of children and no parents met none of the 24hMB guideline, mainly due to MVPA levels, which averaged 133.2 min per day for mothers and 131.4 min for fathers.

With no significant differences between genders, 25.2% of daughters and 26.7% of sons (or 71.7% of daughters and 78.1% of sons) met all 3 (or a combination of ≥ 2) 24hMB guidelines, but with different contributions from mothers and fathers.

In daughters only, the prevalence of excess body weight in fathers significantly reduced the odds ratio of achieving the recommended SB/a combination of ≥ 2 24hMB guidelines (*p* = 0.03/0.003) (Table 2). Maternal excess body weight significantly reduced the odds ratio of fulfilling the recommended amount of ST in daughters (*p* = 0.04) and sons (*p* = 0.002), and furthermore, significantly lowered the odds of achieving the recommended amount of MVPA in daughters (*p* = 0.03) and a combination of ≥ 2 WHO 24hMB guidelines in sons (*p* = 0.03). In addition, university maternal education significantly contributed to compliance with the recommended amount of ST in both daughters (*p* = 0.03) and sons (*p* = 0.02) and to achieving a combination of ≥ 2 24hMB guidelines in daughters (*p* = 0.04). Mothers’ adherence to a combination of ≥ 2 24hMB guidelines significantly increased the odds of their daughters meeting the same combination of the 24hMB guidelines (*p* = 0.006) (Table 2).

Active participation in OPA during leisure time was the only offspring-related variable that significantly helped children achieve the MVPA guideline, daughters met any combination of at least 2 of the 3 WHO 24hMB guidelines, and sons met the ST-related guideline (Table 2). 51.5% of sons and 43.0% of daughters from couple parenting families actively engaged in OPA in their leisure time.

## Discussion

A key finding of the study was that, on average, ¼ of children aged 3–10 years (25.2% of daughters and 26.7% of sons), or nearly ¾ of offspring (71.7% of daughters and 78.1% of sons) met either all 3 WHO 24hMB guidelines or at least a combination of any 2. There were no significant differences by gender or age of the children, but notable differences were observed in the contributions of mothers and fathers in couple-parented families.

Not only was there no apparent gender preference in offspring’s compliance with the 24hMB guidelines linked to mothers, as was observed with fathers, but mothers’ non-excessive body weight, higher level of education and meeting ≥ 2 WHO 24hMB were more frequently and significantly associated with the likelihood of offspring achieving the 24hMB guidelines than those of fathers. Greater assistance from mothers in meeting the 24hMB guidelines is consistent with findings that mothers tend to structure, lead, teach, and engage in empathic conversations, whereas fathers are more inclined to engage in physical play, adopt a peer-like role, follow the child’s lead, and offer challenges [37]. While parents have been shown to play a greater role in helping daughters than sons adhere to the 24hMB guidelines, this contrasts with findings from a study (15) of 8–9-year-old English children, in which parents were more likely to encourage sons to be more physically active than daughters. Overall, mothers had a greater tendency than fathers to encourage their children to be physically active during the week [15]. However, children were most physically active when both parents shared a supportive role [15]. In our study, it is very likely that a common or complementary role of fathers and mothers in promoting the 24hMB guidelines compliance in their offspring can be found through their offspring’s participation in OPA.

Regular active participation in OPA during leisure time significantly helped daughters and sons meet the recommended levels of MVPA. It also supported sons in meeting the recommended ST guidelines, and daughters in achieving any combination of at least 2 or more components of the 24hMB guidelines, regardless of their age or body weight. In line with a Danish prospective study [38], regular active participation in OPA has been shown to contribute to higher overall levels of MVPA and to meeting the daily recommendation of at least 60 min of MVPA in both girls and boys [38]. In addition, participation in OPA during leisure time positively supports meeting additional components of the 24hMB guidelines, thus helping to “harmonize” the movement behaviors of young children. This finding is consistent with a previous study related to the FAMIPASS project, which confirmed a significantly higher likelihood of 3–10-year-old children from family days (mother-child or father-child) achieving a combination of at least 2 of 3 24hMB guidelines when participating in OPA during leisure time, compared with non-participants in OPA [39]. Because this study analyzed families with young children, it is likely that in many cases parents accompanied their children to and from OPA in their free time. However, it was not possible to determine from family diary entries which parent primarily provided logistical support to their children. Nevertheless, given that the research included family triads whose members fully completed the seven-day monitoring, we hypothesize that both parents may have had a more equal role in providing logistical support to their children in OPA.

This study of families with couple parenting provides a ‘springboard’ for examining parental support for the 24hMB guidelines in single-parent and socially disadvantaged families, to determine whether children from these families lag behind those from couple-parent families in meeting the 24hMB guidelines. In follow-up research, the 24hMB patterns of parents and children in single-parent families will be examined, especially since 20,800 married couples, representing 40% of all married couples, divorced in Czechia in 2024. Divorces were most common after 4 to 7 years of marriage and 58% of divorced couples had a minor child in common. This means that a total of 19,300 minor children were affected by divorce [40].

### Strengths and limitations of the study

The strength of the study is the detailed assessment of the main movement components within the 24-hour cycle through continuous instrumental monitoring that captures the typical school/work routines of all family members. The study’s strength also includes the effort to represent families from all socioeconomic backgrounds in urban and rural areas of Bohemia, Moravia and Silesia. Another strength of this study is the fact that 90% of the final sample of families agreed to participate in follow-up monitoring of 24hMB in 2025–2026, making it easier to confirm or refute the conclusions. A notable limitation of the study is its reliance on participants’ interest, willingness, and unpaid voluntary participation in the research. It can reasonably be assumed that participation in the research was confirmed by those with intrinsic motivation and interest, who may have been more prone to healthy behaviors than those without interest in participating in the research. However, in terms of the prevalence of overweight/obesity as calculated by BMI, the sample of parents we analyzed did not differ from the average values of the adult population in this age group [41]. 18% of the families had multiple children participating in the study, resulting in one parent being included multiple times in the analyses examining the parent–child relationship in adherence to 24hMB guidelines. However, a subgroup sensitivity analysis indicated no significant differences in the odds ratios between groups when a parent was included multiple times versus only once. Another limitation is the lack of identification of the reasons for incomplete families (i.e., widowhood, divorce, or religious beliefs) due to concerns that collecting this sensitive information might reduce the number of families willing to participate in the study. The cross-sectional research design does not, of course, allow us to formulate the observed relationships causally, but with the support of theoretical foundations [3–5] we interpret the relationships in the parent-child direction, not the other way around.

## Conclusions

- 25.2% of daughters and 26.7% of sons, and almost 75% the offspring (71.7% of daughters and 78.1% of sons) met all 3 of the WHO 24hMB guidelines for sleep, PA and SB, or at least a combination of any 2 of the 3 guidelines, with no significant differences between gender or age, but with very different contributions from mothers and fathers from couple-parenting families.
- Mothers’ non-excessive body weight, higher level of education and meeting ≥ 2 WHO 24hMB recommendations, compared with those of fathers, are significantly associated with children’s adherence to the WHO 24hMB guidelines in two-parent households. These findings suggest that mothers play an important role in shaping the daily 24hMB that supports their children’s health.
- Regular active participation in OPA significantly assisted both daughters and sons in meeting the recommended MVPA; it also supported sons in meeting the recommended ST and daughters in achieving any combination of at least 2 or more 24hMB guidelines, regardless of children’s age or body weight level.
- A possible indicator of a healthy lifestyle in families with young children seems to be non-excessive parental body weight (especially in mothers), which helps children to meet the 24hMB guidelines (especially in reducing excessive daily ST).

## Data Availability

The datasets analyzed during the current study are not publicly available because this study is part of a longitudinal research project. The baseline phase data are subject to privacy and ethical restriction as participants in this study have signed consent forms stipulating that individual data will not be made publicity available until five years after the completion of the follow-up phase.Aggregated data can be provided by the corresponding author of the study based on a professional scientific query with a clear objective and focus for which the data are queried (10.5281/zenodo.15674732).

## Abbreviations

BMI: Body Mass Index
cm: centimeter
CI: Confidence interval
FAMIPASS: Family physical activity, Sedentary behaviour and Sleep Study
m: meter
M: mean
MVPA: Moderate-to-vigorous physical activity
N: number
OPA: Organized physical activity
OR: Odds ratio
p: level of significance
PA: Physical activity
r: Pearson correlation
Ref.: Reference group
SB: Sedentary behavior
SES: Socioeconomic status
SD: Standard deviation
ST: Screen time
WHO: World Health Organization
24hMB: 24-hour movement behavior
χ^2^: Pearson Chi-square test
%: percentages
